# TNF-*α* Autocrine Feedback Loops in Human Monocytes: The Pro- and Anti-Inflammatory Roles of the TNF-*α* Receptors Support the Concept of Selective TNFR1 Blockade *In Vivo*

**DOI:** 10.1155/2016/1079851

**Published:** 2016-09-22

**Authors:** Jennie M. Gane, Robert A. Stockley, Elizabeth Sapey

**Affiliations:** ^1^Department of Clinical and Experimental Medicine, University of Birmingham, Edgbaston, Birmingham, UK; ^2^Lung Investigation Unit, University Hospital Birmingham NHS Foundation Trust, Edgbaston, Birmingham, UK

## Abstract

Selective TNFR1 blockade in inflammatory diseases is emerging as a clinical strategy. We studied the roles of the two TNF-*α* receptors, TNFR1 and TNFR2, in human monocytes, the principal producer of TNF-*α* and central to many TNF-*α* driven diseases. We hypothesised that TNF-*α* has pro- and anti-inflammatory effects on monocytes, occurring differentially via TNFR1 and TNFR2. Monocytes were isolated from healthy human subjects and exposed to LPS, plus/minus the addition of blocking antibodies to TNF-*α* or its receptors. Pro- and anti-inflammatory cytokine production was quantified using real-time PCR and ELISAs. Cell surface expression of TNFR1/2 was measured by flow cytometry. We demonstrated that monocytes vary in the expression patterns of TNFR1 and TNFR2. Autocrine binding of TNF-*α* led to sustained upregulation of proinflammatory cytokines via TNFR1. In contrast, autocrine binding via TNFR2 upregulated the* anti*-inflammatory cytokine, IL-10, without proinflammatory effect. TNFR2 was responsible for binding soluble TNF-*α* secreted by monocytes, clearing the cytokine from the pericellular environment. TNFR1 blockade did not change the cell surface expression of TNFR2, leaving this receptor free to upregulate IL-10. These novel results support the concept of selective TNFR1 blockade* in vivo* in order that positive anti-inflammatory effects of TNF-*α* can be retained via TNFR2 ligation.

## 1. Introduction

TNF-*α* is classically regarded as a proinflammatory cytokine, playing an important role in the pathophysiology of inflammatory diseases, such as rheumatoid arthritis and inflammatory bowel disease [1]. Anti-TNF-*α* molecules, which block both soluble and membrane bound TNF-*α*, are effective in inflammatory arthropathies [2, 3] and inflammatory bowel disease [4, 5]. Despite this, 30% of patients with these diseases do not respond to anti-TNF-*α* agents and the reasons are unclear [1]. Intriguingly, some patients receiving anti-TNF-*α* therapy who had multiple sclerosis as a comorbidity developed exacerbation of their neurological disease, suggesting a proinflammatory consequence of generic TNF-*α* blockade in certain circumstances [6, 7].

Monocytes are the principal source of TNF-*α* in humans and are believed to be central in many diseases, including inflammatory bowel disease, arthropathies, and septic shock [1, 8]. Monocytes are also one of the few cells that express both TNF-*α* receptors in humans; TNFR1 is expressed on most human cells whereas TNFR2 is found only on immune cells and vascular endothelial cells [9]. TNFR1 is historically considered as the receptor through which the majority of the proinflammatory effects are elicited [8, 10]. The role of TNFR2 in general and its intracellular signalling pathways is less clear. Studies using murine knockout models suggest it plays a predominantly anti-inflammatory role, completely or partially protecting against experimentally induced arthritis, encephalitis, and heart failure [8], and it may have an important role in virus elimination [11]. However, in some studies, a proinflammatory role has also been suggested, as TNFR2 knockout mice developed less emphysema in response to cigarette smoke [12].

The importance of characterising the respective roles of TNFR1 and TNFR2 in human cells is being increasingly recognised. If TNFR1, ubiquitously expressed on almost all cell types, is the “proinflammatory” receptor, with TNFR2 responsible for a more immunomodulatory role, selective TNFR1 blockade would seem a more appropriate strategy in chronic inflammatory diseases. Indeed, a phase one study of Atrosab, a humanized monoclonal antibody that specifically blocks TNFR1, has demonstrated an acceptable safety profile and phase two proof-of-concept trials in rheumatoid arthritis and psoriasis are planned for 2016 [13]. In addition, another monoclonal antibody targeting TNFR1, GSK1995057, has been tested in healthy volunteers with an associated reduction in proinflammatory mediators in bronchoalveolar lavage fluid in response to LPS [14]. Conversely, recent studies in platelets from rheumatoid arthritis have shown proinflammatory effects of TNF-*α* signalling via TNFR2, specifically upregulation of the adhesion molecule P-selectin leading to platelet-neutrophil complex formation [15].

We hypothesised that TNF-*α* signalling in monocytes has both pro- and anti-inflammatory effects, occurring differentially through TNFR1 and TNFR2. Our experimental aims were as follows: to study the respective roles of the two receptors on autocrine regulation of pro- and anti-inflammatory cytokine production, to assess the expression patterns of both receptors on monocytes, and to study the effects of TNF-*α* on their cell surface expression pattern.

## 2. Materials and Methods

### 2.1. Study Subjects

Peripheral blood monocytes were obtained from 19 healthy subjects (12 male) who were nonsmokers and not receiving regular medication. The median age was 30 years (range: 21 to 45) and all subjects provided informed consent. The studies had received approval from a research ethics committee (Research and Ethics Committee number 3359a).

### 2.2. Isolation of Blood Monocytes

Whole blood was collected into heparinised tubes, layered over Lymphoprep*™* (Axis Shield, Stockport, UK), and centrifuged to obtain a buffy coat layer of PBMCs. The cells were resuspended in sterile PBS containing 2 mM EDTA and 0.1% BSA and CD14+ CD16− monocytes were extracted using the Dynabeads® Untouched*™* Human Monocytes kit (Life Technologies, Paisley, UK), following the product protocol. The monocytes (>95% viable by exclusion of trypan blue) were resuspended in culture medium (sterile RPMI medium 1640; Sigma Chemicals Ltd., Poole, UK) supplemented with 10% foetal calf serum, 10% L-glutamine, and 10% penicillin V and streptomycin. Throughout all stages of the isolation, the cells were kept on ice to minimise cellular activation.

### 2.3. Cell Culture/Stimulation

Cell culture was conducted in 12 or 24-well tissue culture plates (Costar, Fisher Scientific, Loughborough, UK) at 37°C and in 5% CO_2_. Monocytes were plated at a concentration of between 0.25 and 0.45 million per mL of culture medium. The number of replicates used is specified in individual experiments.* Salmonella* Enteritidis derived LPS (100 ng/mL; Sigma Chemicals Limited, Poole, UK) was used as the TNF-*α* stimulant. The blocking mAbs used in relevant experiments are shown in Table 1 and are marketed as preventing binding of each relevant protein to its usual ligand, thereby blocking the usual function of that protein. Each mAb was added at a concentration of 10 *μ*g/mL to 1 mL of culture medium per well (except in Figure 4 where 25 *μ*g/mL of TNF-*α* mAb was used to ensure that all sTNF-*α* and mTNF-*α* were blocked), following initial concentration response experiments. An irrelevant mouse IgG_1_ antibody was used as a control in validation experiments to exclude any nonspecific effect of the mAbs (data not shown). Culture plates containing cells incubated with mAbs were gently agitated for 20 minutes prior to the addition of LPS.

### 2.4. Measurement of Inflammatory Mediators in Cell Culture Supernatants

Concentrations of the mediators were measured in monocyte supernatants using specific ELISAs according to manufacturer instructions and read using a Synergy HT microplate reader (Biotech, GMI, Ramsey, USA). All samples and standards were run in duplicate and the average was taken as the sample result. Manufacturers' details and lower and upper limits of quantification are given in Table 1.

### 2.5. RNA Isolation, Reverse Transcription, and Real-Time Quantitative PCR

Adherent cells were pelleted and stored in RNA*later*® (Life Technologies, Paisley, UK) at −80°C for future study. Messenger RNA was extracted from each cell pellet using the Isolate RNA Minikit (Bioline, London, UK) and adequate quantity and purity of extracted RNA were determined using a Nanodrop 2000c spectrophotometer (Fisher Scientific, Loughborough, UK). RNA samples were reverse transcribed to cDNA using a High Capacity RNA-to-cDNA Kit (Life Technologies, Paisley, UK) and real-time quantitative PCR carried out for each gene of interest using TaqMan*™* gene expression assays (Life Technologies, Paisley, UK), as listed in Table 1. All assays were conducted as described previously [16]. GAPDH was found to be stably expressed in a random selection of samples and was therefore used as the normalising gene in the current studies (data not shown). The 2^−ΔCT^ formula was used to calculate the relative expression of mRNA of the genes of interest [17].

### 2.6. Analysis of TNF-*α* Receptors 1 and 2 Expression on the Surface of Monocytes

Monocytes (0.25 × 10^6^ per sample) underwent flow cytometry either immediately after isolation or after a relevant period in culture and were labelled with fluorophore-labelled mAbs to TNFR1 and TNFR2, as described in [18]. Control samples labelled with isotype matched control mAbs were run simultaneously to control for nonspecific binding. Full antibody details are provided in Table 1. Positively labelled samples were run in duplicate where specified and the mean result was taken. Doublet events were excluded. MFI results are expressed as the ratio of the intensity of cells labelled with the flow cytometry antibody of interest to those incubated with isotype control flow cytometry antibodies.

### 2.7. Statistical Analysis

Data are presented as median and interquartile range/range or mean and standard deviation where percentages are presented. Friedman's test was employed to detect overall differences between experimental conditions or time points, followed by* post hoc* pairwise comparison testing if appropriate. Individual pairwise comparisons were tested using a Wilcoxon Signed-Rank Test. Area under the curve calculations were made using the trapezoid method. Statistical significance was set at *p* ≤ 0.05. Two tailed *p* values were used unless otherwise specified. One-tailed *p* values were employed where previous experimental work provided a clear expectation of effects. Data was analysed using the SPSS statistical program (version 22.0, Chicago, USA).

## 3. Results

### 3.1. Autocrine Regulation of Monocyte Inflammatory Cytokine Production by TNF-*α*

**Figure 1:**
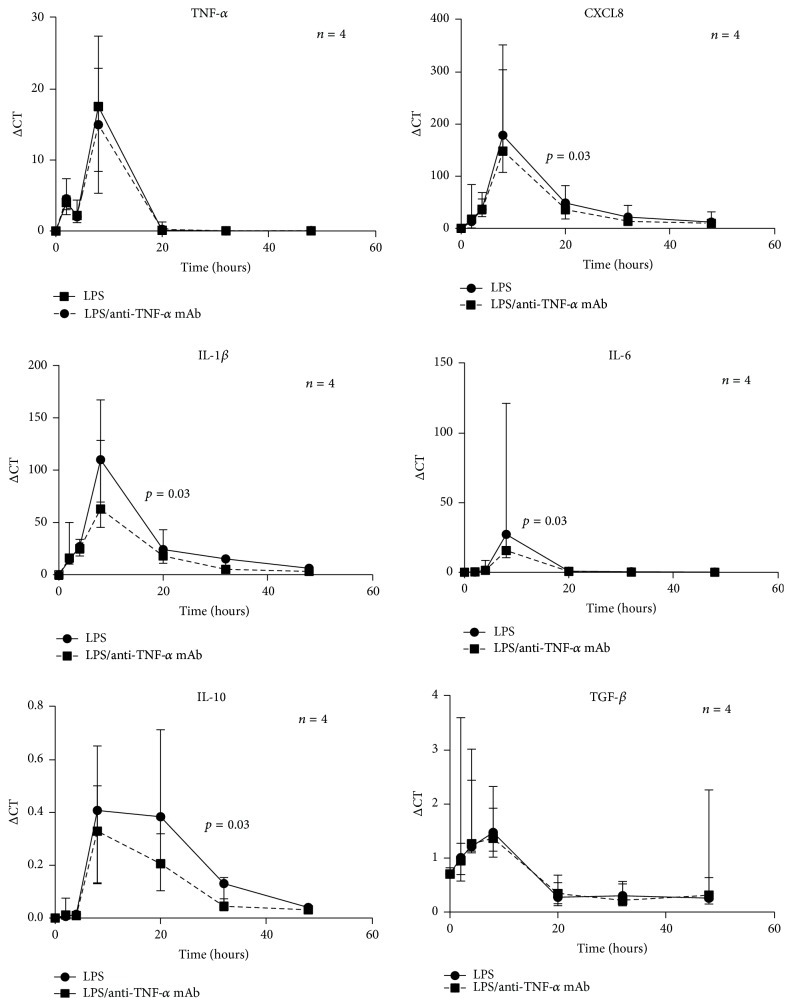
Effect of TNF-*α* mAb on pro- and anti-inflammatory cytokine mRNA expression in LPS-stimulated monocytes. Each figure illustrates a time course experiment showing cytokine mRNA expression by monocytes stimulated with LPS (100 ng/mL) ± TNF-*α* mAb (10 *μ*g/mL). Data points are expressed as median (range). AUC values were compared using the Wilcoxon Signed-Rank Test. *p* values are one-tailed and are shown if significant. A significant reduction in AUC in the presence of TNF-*α* mAb was observed for CXCL8, IL-1*β*, IL-6, and IL-10 (*p* = 0.03). The experiments represent data from 4 subjects. Time points were conducted singly in culture wells and the resultant cellular material in duplicate for subsequent real-time PCR.

To determine whether autocrine binding of TNF-*α* had a positive feedback effect on monocytes, cells (from *n* = 4) were stimulated with LPS in the presence or absence of TNF-*α* mAb.  Figure 1 shows the time course profiles of mRNA expression of TNF-*α*, CXCL8, IL-1*β*, IL-6, IL-10, and TGF-*β* after stimulation with LPS and LPS in the presence of TNF-*α* mAb.  Table 2 details median AUC values for each cytokine time course profile for both experimental conditions. Significantly lower AUC values in LPS-stimulated monocytes cultured in the presence of TNF-*α* mAb were observed for CXCL8, IL-1*β*, IL-6, and IL-10 (*p* values are shown in Figure 1 if significant). TNF-*α* and TGF-*β* mRNA expression were not affected by blockade of secreted TNF-*α* mAb. To test the hypothesis that the effect of TNF-*α* blockade would also influence the secreted protein, the concentration of one mediator, CXCL8, was determined in the supernatants from the experiments shown in Figure 1 (*n* = 4) at each time point by ELISA. A significant reduction in CXCL8 in the LPS-stimulated cell supernatants was observed from 32 hours onwards in the presence of TNF-*α* mAb, from 172.7 ng/mL (range: 150.6 to 237.0 ng/mL) to 138.3 ng/mL (range: 122.3 to 159.4 ng/mL) at 32 hours (*p* = 0.03).

### 3.2. The Role of Individual Receptors

**Figure 2:**
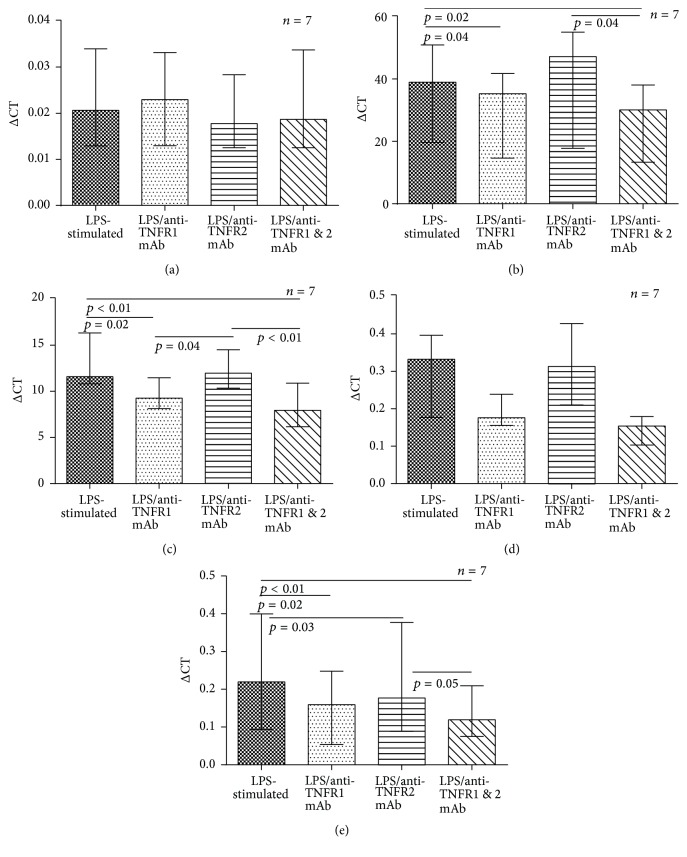
Effect of selective TNF-*α* receptor blockade on cytokine mRNA expression. Monocytes were stimulated with 100 ng/mL of LPS (positive control) for 20 hours ± TNFR1/TNFR2 mAb/both (10 *μ*g/mL), and cytokine mRNA expression was measured. Each column shows median (IQR) values. Overall differences between experimental conditions for each cytokine were assessed with Friedman's test and* post hoc* pairwise comparisons conducted if appropriate (*post hoc p* values are shown in the figures). (a) TNF-*α*: no overall difference observed. (b) CXCL8: Friedman's test *p* = 0.03. (c) IL-1*β*: Friedman's test *p* < 0.01. (d) IL-6: no overall difference observed. (e) IL-10: Friedman's test *p* < 0.01. The experiments represent data from 7 subjects. Experimental conditions were conducted singly in culture wells and the resultant cellular material used in duplicate for subsequent real-time PCR.

To determine the role of individual TNF-*α* receptors following autocrine binding of TNF-*α*, monocytes (from *n* = 7) were cultured following stimulation with 100 ng/mL LPS in the presence or absence of 10 *μ*g/mL TNFR1 mAb or TNFR2 mAb or both. No effect was observed for TNF-*α* mRNA expression (Figure 2(a)). However, significant reductions in expression of CXCL8, IL-1*β*, and the anti-inflammatory cytokine IL-10 were observed when TNFR1 alone was blocked (Figures 2(b), 2(c), and 2(e)) and a similar trend (though not statistically significant) was observed for IL-6 (Figure 2(d)). TNFR2 blockade alone had no effect on proinflammatory gene expression but did result in a reduction in IL-10 expression (see Figure 2(e)) and dual receptor blockade led to greater reduction in IL-10 mRNA than blocking TNFR2 alone.

### 3.3. TNFR1 and TNFR2 Surface Expression on Monocytes

**Figure 3:**
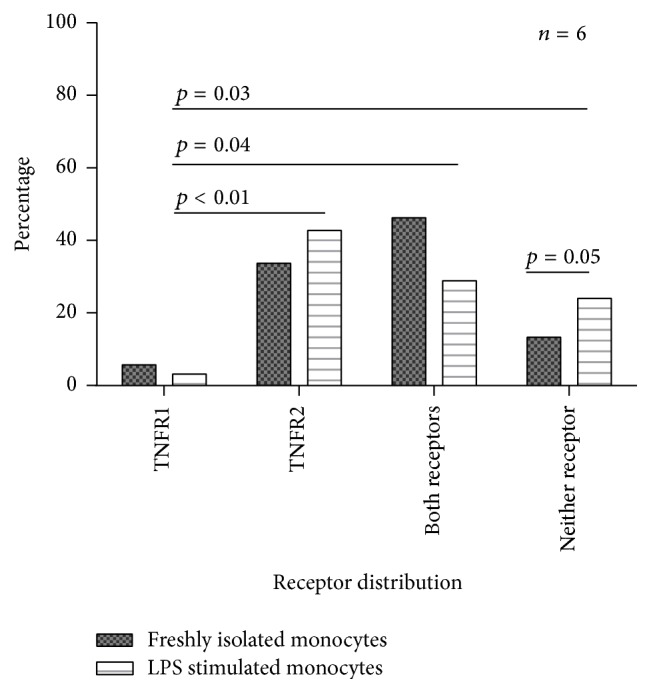
Pattern of TNF-*α* receptor expression on monocytes. Each column shows the mean percentage of monocytes (with SD bars) expressing each of the four possible cell surface receptor combinations, in freshly isolated cells and after stimulation with 100 ng/mL of LPS for 20 hours. Overall differences in receptor distribution subtype for each of the two conditions were assessed with separate Friedman's tests and* post hoc* pairwise comparisons conducted if appropriate. In freshly isolated unstimulated monocytes, no overall difference was observed. In monocytes stimulated for 20 hours with 100 ng/mL of LPS, Friedman's test was significant (*p* = 0.02). Significant* post hoc p* values are shown in the figure and were significant for comparisons with monocytes expressing TNFR1 only. Comparisons for each of the four receptor distribution subtypes between freshly isolated and LPS-stimulated monocytes were conducted with Wilcoxon Signed-Ranks Testing and demonstrated an increase in the percentage of monocytes expressing neither receptor in LPS-stimulated cells. The experiments represent data from 6 subjects. Experimental conditions for freshly isolated monocytes were conducted singly and for LPS-stimulated monocytes in duplicate (due to limitations to cell numbers available).

Current therapeutic strategies to target TNF-*α* affect signalling through both TNFR1 and TNFR2.  Figure 3 shows the distribution of the pattern of receptor expression on freshly isolated monocytes and monocytes which had been stimulated with LPS. There appeared to be four distinct subpopulations of cells in freshly isolated monocytes with some expressing TNFR1 alone (5.8%  ± 5.0%), TNFR2 alone (34.0%  ± 34.0%), both (46.7%  ± 34%), or neither receptor (13.5%  ± 13.3%). However, both before and following stimulation, most cells expressed either TNFR2 alone or both receptors. A small but significant increase in the percentage of monocytes expressing neither receptor was observed in LPS-stimulated cells compared to freshly isolated monocytes. Raw data broken down by subject for freshly isolated monocytes is shown in Table 3 and illustrates the high degree of intrasubject variation in expression pattern observed.

### 3.4. The Autocrine Effect of TNF-Alpha on Cell Surface TNFR1 and TNFR2

We wished to determine whether TNF-*α* affects the expression of its own receptors, in addition to upregulating cytokines. To investigate this, monocytes were stimulated with LPS with and without an excess of either TNF-*α* mAb (to prevent cell-derived TNF-*α* binding to the cell via either receptor) or a mAb specific to either of the two TNF-*α* receptors (to ensure TNF-*α* signalling occurred solely via one of its two receptors).

**Figure 4:**
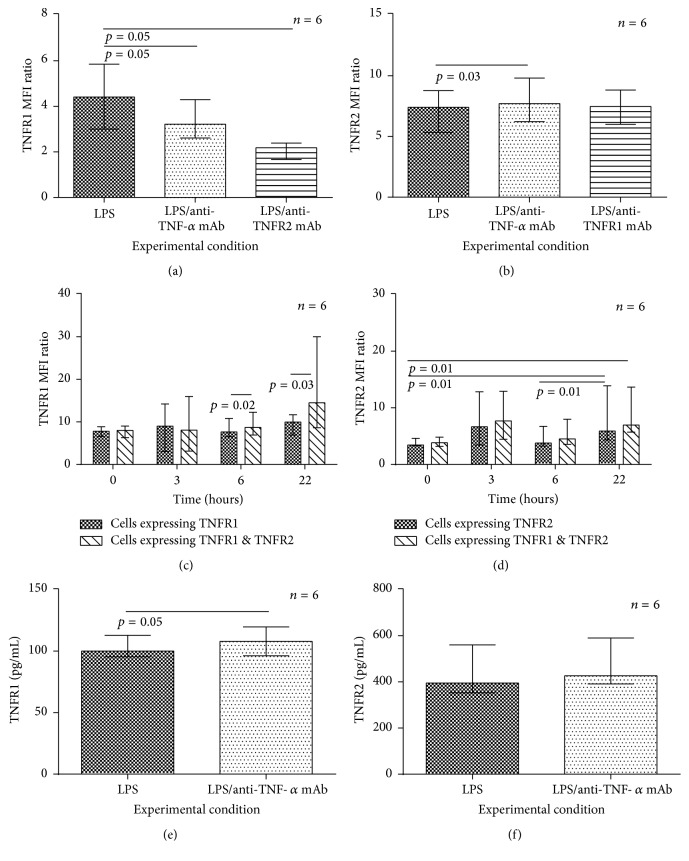
Autocrine effects of TNF-*α* on TNF-*α* receptor cell surface expression and soluble receptor concentration. In (a) and (b), the surface expression of TNFR1 and TNFR2 on monocytes stimulated with LPS (100 ng/mL) ± TNF-*α* mAb or selective TNF-*α* receptor blockade (25 *μ*g/mL) for 20 hours was determined (e.g., TNFR1 expression was determined in monocytes where TNF-*α* signalling via TNFR2 had been prevented with a blocking mAb). Concentrations were compared to LPS-stimulated cells, using a Wilcoxon Signed-Ranks Test. In (c) and (d), time course profiles of TNFR1 MFI and TNFR2 MFI, respectively, are shown in LPS-stimulated monocytes expressing one or both types of receptor. No overall difference was observed for time course profiles of TNFR1 expression in cells expressing solely TNFR1 (Friedman's *p* = 0.33) nor cells expressing both receptors (Friedman's *p* = 0.09). Friedman's test was significant for TNFR2 expression in cells expressing TNFR2 solely (*p* = 0.02) and cells expressing both receptors (*p* = 0.02). Significant* post hoc* pairwise comparisons *p* values are shown in the figures. Differences in MFI at each time point between cell types were assessed with a Wilcoxon Signed-Ranks Test. In (e) TNFR1 and (f) TNFR2, the columns show the concentration in the supernatant of LPS-stimulated monocytes (for 22 hours) ± TNF-*α* mAb (10 *μ*g/mL). Concentrations were compared to the positive control, LPS-stimulated cells, using a Wilcoxon Signed-Ranks Test. Columns in all figures show median (IQR) values. The experiments represent data from 6 subjects. Experimental conditions/time points for flow cytometry analyses were conducted in duplicate (a–d) with the exception of freshly isolated monocytes (time: 0 hours) in (c) and (d) due to limitations to cell numbers available. Experimental conditions in (e) and (f) were conducted in culture wells in duplicate and in each sample run in duplicate on the subsequent ELISA.

**Figure 5:**
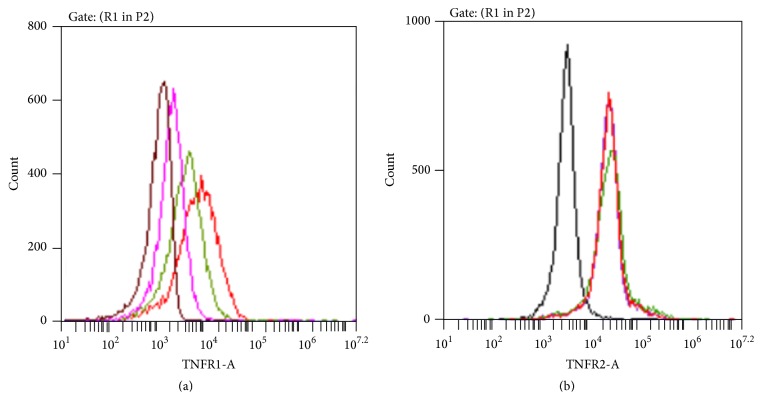
An example of flow cytometry overlay histograms. Overlay histograms are shown for gated monocytes from one subject. (a) Four histograms are shown, with fluorescence intensity in the PE channel on the *x*-axis and cell count on the *y*-axis. The black histogram represents LPS-stimulated monocytes incubated with a PE-labelled isotype control antibody. The red histogram represents monocytes stimulated with LPS and labelled with PE-TNFR1 mAb. Green and pink histograms represent LPS-stimulated monocytes incubated in the presence of TNF-*α* mAb or TNFR2 mAb, respectively, and then labelled with PE-TNFR1 mAb. The altered positions of the histograms illustrate the effect of TNF-*α* or TNFR2 blockade on subsequent TNFR1 expression. (b) Four histograms are shown, with fluorescence intensity in the FITC channel on the *x*-axis and cell count on the *y*-axis. The black histogram represents LPS-stimulated monocytes incubated with a FITC-labelled isotype control antibody. The red histogram represents monocytes stimulated with LPS and labelled with FITC-TNFR2 mAb. Green and pink histograms represent LPS-stimulated monocytes incubated in the presence of TNF-*α* mAb or TNFR1 mAb, respectively, and then labelled with FITC-TNFR2 mAb. Red, green, and pink histograms overlay each other exactly illustrating that in this subject's monocytes there was no effect of TNF-*α* or TNFR1 blockade on subsequent TNFR2 expression. Each of the three experimental conditions was conducted alongside samples subjected to the same conditions but incubated with an isotype control flow cytometry antibody. As all isotype control histograms overlaid each other, only one of three is shown in each graph for ease of visualisation.

Figures 4(a) and 4(b) show that blockade of TNF-*α* led to a significant decrease in TNFR1 surface expression and conversely a small but significant increase in TNFR2. In addition, Figure 4(a) shows that TNFR1 membrane expression was decreased further when TNFR2 was blocked rather than TNF-*α* itself. The results suggest that there is a positive feedback loop in monocytes, where TNFR1 expression is increased following signalling through TNFR2 by TNF-*α*. An example of flow cytometry overlay histograms from one subject are shown in Figure 5.

Given the findings in Figure 4(a), we hypothesised that we would observe an increase in the TNFR1 MFI ratio over time in stimulated monocytes expressing both TNFR1 and TNFR2, greater than any increase in monocytes solely expressing TNFR1, in which TNF-*α* autocrine feedback via TNFR2 could not occur.  Figure 4(c) shows a trend for an increase over time of TNFR1 expression in monocytes expressing both receptors after LPS stimulation (Friedman's test *p* = 0.09) and shows that by 6 and 22 hours after stimulation TNFR1 MFI ratio was significantly greater in the monocytes expressing both TNFR1 and TNFR2 than in those expressing TNFR1 only. In contrast, although there was a significant increase in TNFR2 expression over time in monocytes expressing TNFR2 alone or both receptors, there was no difference between those two monocyte types (Figure 4(d)).

To determine if the reduction in cell surface TNFR1 following TNF-*α* blockade was due to increased shedding of the receptor into the pericellular environment, soluble TNFR1/TNFR2 concentrations were measured in cell-free supernatants from monocytes (stimulated with LPS with and without a TNF-*α* mAb). Figures 4(e) and 4(f) show TNF-*α* mAb caused a small but significant increase in soluble TNFR1 consistent with an autocrine effect but had no effect on soluble TNFR2 concentration.

### 3.5. Autocrine Binding of Soluble TNF-*α* Occurs Predominantly via TNFR2 in Monocytes Cultured in Isolation

**Figure 6:**
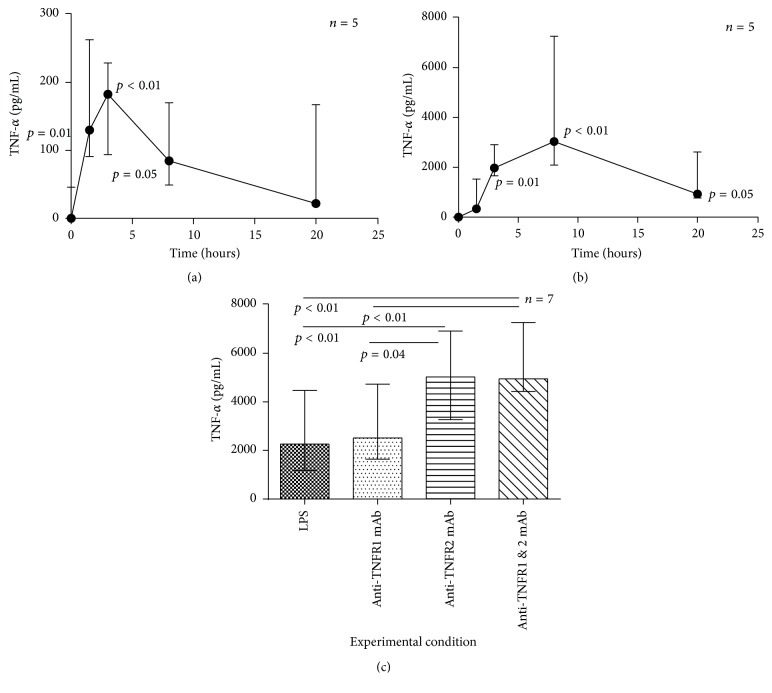
Autocrine binding of sTNF-*α* secreted by monocytes. Time course profiles for monocytes cultured alone (a) or with 100 ng/mL of LPS (b) are shown. Each datum point is the median value (range). (a) Friedman's test: *p* < 0.01. Significant post hoc pairwise comparisons of each time point to time zero are shown in the graphs. (b) Friedman's test: *p* < 0.001. Significant post hoc pairwise comparisons of each time point to time zero are shown in the graphs. (c) Monocytes were cultured with 100 ng/mL of LPS for 20 hours ± 10 *μ*g/mL of TNFR1 mAb or TNFR2 mAb or both mAbs. Each column shows median and IQR values. Friedman's test: *p* < 0.001.* Post hoc* pairwise comparisons *p* values are shown in the figures. The experiments represent data from 5 subjects (a and b) or 7 subjects (c). Time points in (a) and (b) were conducted in culture wells singly and experimental conditions in (c) in duplicate. Each sample was run in duplicate on the subsequent ELISAs.

It was noted in early experiments that the concentration of sTNF-*α* in the culture medium of monocytes initially increased, in the absence of (Figure 6(a)) and in response to LPS (Figure 6(b)), but was followed by a subsequent decrease.

The TNF-*α* ELISA detects both free and soluble receptor-bound cytokine indicating that the fall in detectable sTNF-*α* was not due to binding to its soluble receptors but could represent degradation or exclusion from the supernatant by binding to cell surface receptors.  Figure 6(c) shows TNF-*α* concentration in the cell-free supernatant of monocytes stimulated with LPS in the presence or absence of selective TNF-*α* receptor blockade. Blockade of TNFR1 did not lead to an increase in sTNF-*α*, whereas blockade of TNFR2 did, indicating the decrease in sTNF-*α* over time was likely due to binding to TNFR2.

## 4. Discussion

The current series of experiments provides novel data concerning TNF-*α* autocrine feedback loops, the respective roles of TNFR1 and TNFR2 in human monocytes, and the finding that human monocytes have a variable expression pattern of TNF-*α* receptors.

Global TNF-*α* blockade* in vivo* is generally anti-inflammatory [8] but is associated with the abrogation of both pro- and anti-inflammatory cytokine production in monocytes. Signalling for proinflammatory mediators in monocytes occurred via TNFR1 whereas anti-inflammatory IL-10 upregulation occurred via either TNFR1 or TNFR2, with an additive effect of dual receptor ligation. Data from the current work suggests that on average over 80% of monocytes studied expressed TNFR2, whilst approximately 50% expressed TNFR1 (with a high degree of intrasubject variation). Global TNF-*α* blockade will therefore have a significant effect on both pro- and anti-inflammatory signalling from monocytes, whilst TNFR1 blockade would provide a predominantly anti-inflammatory effect.

Previous authors studying the respective roles of the two receptors in human cells have been limited by the use of exogenous sTNF-*α* or alternatives such as mutant forms of TNF-*α* which can activate TNFR2 and/or cell lines where mTNF-*α* has been overexpressed [19–21]. Whilst useful, these models may not resemble naturally occurring events with mTNF-*α*. In our experimental system, we used LPS as a potent but relevant inducer of TNF-*α* [22] as mTNF-*α* must be produced before its release as sTNF-*α*, providing confidence that signalling could occur through both forms of the cytokine. This makes our experiments more physiological. In addition, the same batch of LPS was used throughout to overcome potential variations between bacterial sources and even batches from the same strain [23] to ensure the best replication of experiments.

IL-10 is also produced by monocytes in response to bacterial products such as LPS [24]. In our study, we saw an additional positive feedback effect of TNF-*α* on IL-10 mRNA expression, illustrating the complexity of feedback loops as IL-10 itself can downregulate TNF-*α* expression [25]. IL-10 mRNA expression was upregulated by TNF-*α* binding to either receptor, additively, and importantly TNFR2 ligation alone induced IL-10 upregulation in the absence of any significant upregulation of proinflammatory cytokines. IL-10 is a key immune-regulatory cytokine with anti-inflammatory effects in many diseases. Monocytes, in particular the dominant CD14+ CD16− monocytes studied here, and other cells of the monocyte lineage are a major source of IL-10 [26]. By blocking the TNF-*α* molecule itself, for example, with infliximab, any autocrine upregulation of IL-10 as a result of TNF-*α* binding might be lost, disrupting the proinflammatory/anti-inflammatory balance, and may explain the adverse events in patients with multiple sclerosis [6, 7].

These findings provide supporting evidence in favour of TNFR1 blockade* in vivo*, in diseases in which TNFR1 signalling has been shown to play the dominant role in pathogenesis, such as inflammatory arthritis and multiple sclerosis [8, 27]. TNFR1 expression is ubiquitous, whereas TNFR2 expression is limited to leukocytes and vascular endothelial cells [9]. Selective blockade of TNFR1 in a clinical setting might therefore ameliorate the proinflammatory effects of TNF-*α*, whilst allowing ongoing beneficial effects of anti-inflammatory IL-10 produced by monocytes via TNFR2 signalling. To this end, our data supports preliminary* in vivo* studies examining the role of selective TNFR1 blockade in an LPS-model designed to simulate acute lung injury [14].

The reduction in sTNF-*α* in the supernatants of our experiments with time is likely to reflect internalisation and degradation of the ligand-receptor complex [28]. The majority of sTNF-*α* binds to TNFR2, an intriguing finding given that mTNF-*α* rather than sTNF-*α* is believed to stimulate TNFR2 [19, 29]. This may represent a previously unrecognised homeostatic mechanism for limiting sTNF-*α* ligation to TNFR1, in the same way that soluble TNF-*α* receptors are able to exclude active TNF-*α* in the circulation [9]. Similar reduction in sTNF-*α* has also been observed in monocyte-derived macrophages, indicating this possible homeostatic mechanism is not restricted to monocytes alone but also to other major cellular producers of TNF-*α* [30]. This concept requires further study as our experiments studied monocytes in isolation and hence did not assess competition for sTNF-*α* binding by TNFR1 on other cell types.

**Figure 7:**
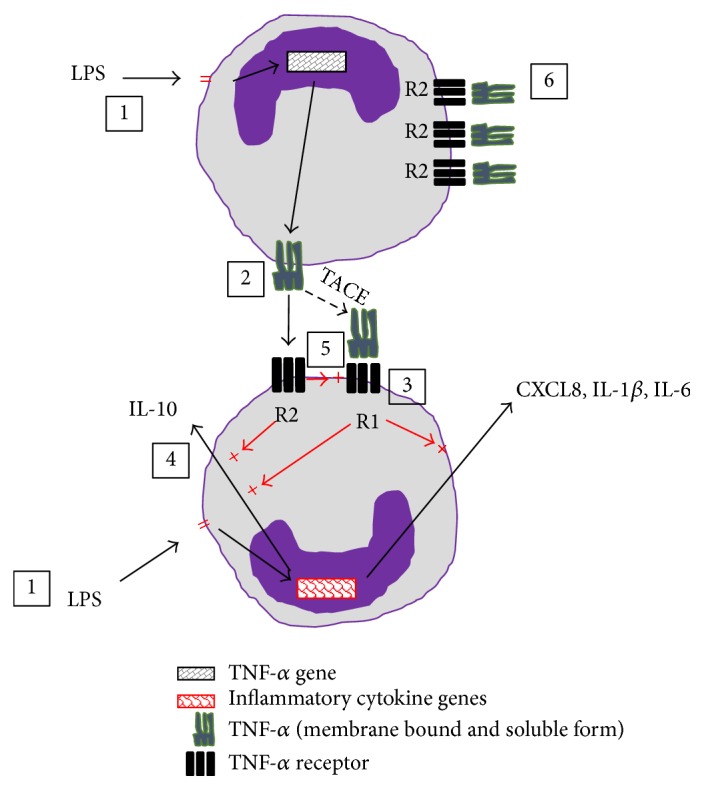
TNF-*α* effects on monocytes via TNR1 and TNFR2. The figure shows two adjacent monocytes. (1) LPS induces both pro- and anti-inflammatory cytokine gene/protein expression. (2) TNF-*α*, induced by LPS stimulation, is expressed at the monocyte surface as mTNF-*α* and cleaved to sTNF-*α* by the enzyme TACE. (3) sTNF-*α* signals in an autocrine/paracrine fashion via TNFR1 to augment the expression of CXCL8, IL1-*β*, IL-6, and IL-10 mRNA and (4) mTNF-*α* via TNFR2 to further augment IL-10 mRNA expression. (5) Autocrine/paracrine binding of membrane TNF-*α* to TNFR2 also upregulates the expression of TNFR1 at the cell surface. (6) TNFR2 binds sTNF-*α*. Selective TNFR1 blockade will switch off TNF-*α* driven proinflammatory cytokine production whilst leaving TNFR2 free to upregulate the immunoregulatory cytokine, IL-10, and possibly clear a proportion of sTNF-*α* from the pericellular environment.

A further novel finding was that signalling via TNFR2 also upregulated the cell surface expression of TNFR1, most likely through increased production of the receptor rather than reduced shedding into the supernatant, as TNF-*α* blockade had only a slight effect on soluble TNFR1 concentration. TNFR1 upregulation did not seem to have an appreciable proinflammatory effect, as blockade of TNFR2 did not reduce proinflammatory cytokine output (through reduction of cell surface TNFR1). The relevance of this increase in TNFR1 secondary to TNF-*α* signalling via TNFR2 on monocytes, which can express both receptors, is unknown but would not negate the clinical efficacy of selectively blocking TNFR1 as any increase in TNFR1 expression on cells by TNFR2 ligation would still be blocked by the TNFR1 mAb. It is also important to note that TNFR1 blockade did not reduce TNFR2 expression on the monocyte surface thereby leaving it available to bind TNF-*α* and induce IL-10 production. The increase in TNFR2 receptors over time is likely to have occurred as a direct result of LPS stimulation (as shown recently [31]).  Figure 7 summarises concepts from the data in pictorial form.

There are some limitations to our study. Firstly, as a practical issue, the monocyte yield is low compared to other leukocytes, and hence the number of cellular functions and experimental conditions we could study at one time was limited, thus requiring separate experiments to generate a global view. Secondly, it was not possible to include a negative control arm to experiments, whereby monocytes were not exposed to LPS. However, validation experiments revealed that monocytes cultured in the absence of LPS became apoptotic, especially at later time points. This meant that obtaining a sufficient number of events for each flow cytometry sample for viable unstimulated cells would not have been possible. Thirdly, if targeted TNFR1 blockade in TNF-*α* disease is to become a viable strategy, it will be necessary to replicate and extend our experiments within the relevant disease cohorts.

Further delineation of the TNFR1 and TNFR2 intracellular signalling pathways in monocytes is also needed. The ability of TNFR2 ligation to induce signalling pathways that initiate IL-10 upregulation in the absence of any upregulation of proinflammatory cytokines, whilst TNFR1 ligation can do both, is of importance in abrogation of the inflammatory/anti-inflammatory balance and hence damage versus resolution. Determining the steps in the pathways should make it possible to separate these effects.

In addition, further work is needed to clarify the role of TNFR2 in monocytes. Whether monocytes that express both TNFR1 and TNFR2 differ substantially in function from those only expressing TNFR1 or TNFR2 and whether expression patterns change in response to a stimulus or as part of an illness are of particular interest. Intriguingly, approximately 15% to 25% of monocytes in both the freshly isolated and stimulated state expressed neither TNFR1 nor TNFR2 and the percentage of monocytes with this subtype increased slightly in response to LPS stimulation. The significance of this finding in the context of monocyte function is unclear and could be investigated further by separating monocytes by TNFR1/TNFR2 status by flow cytometry and tracking changes to receptor expression within each subgroup. Furthermore, it would be informative to examine the functional effects of selective TNFR2 blockade (both soluble and membrane bound). The cell-free supernatants from monocytes cultured in the presence or absence of TNFR2 mAb could be harvested and used as a stimulus for neutrophils or endothelial cells. This would identify if the excess pericellular soluble TNF-*α*, present as a direct result of TNFR2 blockade, could enhance release of neutrophil products such as reactive oxygen species or the expression of adhesion molecules on endothelial cells. This would support the hypothesis that membrane bound TNFR2 has an important homeostatic role in clearing TNF-*α* from the pericellular environment and further support selective TNFR1 blockade* in vivo*.

Lastly, it would be informative to determine whether the imbalance in favour of IL-10 secretion in the presence of TNFR1 blockade has a beneficial effect on macrophage differentiation. IL-10 drives monocytes towards the more immunomodulatory M2 macrophage phenotype (rather than the type 1 proinflammatory M1 phenotype [32]), which in itself could have positive effects* in vivo* in TNF-*α* associated inflammatory diseases. Both macrophage phenotypes can be produced* in vitro* and hence the relative roles of TNFR1 and TNFR2 could be studied by macrophage subtype. However, this may not be relevant* in vivo* as recent studies suggest that macrophages are rarely derived from monocytes [33]. Thus, it would be more relevant to conduct such studies with tissue derived macrophages.

## 5. Conclusions

In susceptible individuals, disordered and excessive TNF-*α* signalling leads to chronic inflammatory disease. TNFR1 is ubiquitous and its ligation by TNF-*α* has widespread proinflammatory effects, whereas TNFR2 expression is limited mainly to leukocytes and its function in humans has been less clear. The data presented here highlights that monocytes vary in their expression patterns of TNFR1 and TNFR2 but support the concept that proinflammatory effects of TNF-*α* occur via TNFR1 whilst TNFR2 predominantly plays an anti-inflammatory role by increasing IL-10 output and removing sTNF-*α* from the pericellular environment. These findings may support the current strategy of selective TNFR1 blockade for TNF-*α* driven chronic inflammatory disease.

## Figures and Tables

**Table 1:** Assays.

mAb	Clone number	Fluorophore	Function	Manufacturer
Mouse IgG_1_ TNF-*α*	28401	N/A	Blocking	R and D Systems
Mouse IgG_1_ TNFRI	16805	N/A	Blocking	R and D Systems
Mouse IgG_1_ TNFR2	22210	N/A	Blocking	R and D Systems
Mouse IgG_1_ isotype control	11711	N/A	Control	R and D Systems
Mouse IgG_1_ TNFR1	16803	PE	Flow cytometry	R and D Systems
Mouse IgG_2A_ TNFR2	22235	FITC	Flow cytometry	R and D Systems
Mouse IgG_1_ isotype control	11711	PE	Flow cytometry	R and D Systems
Mouse IgG_2A_ control	X39	FITC	Flow cytometry	BD Biosciences

ELISA	Minimum detectable dose (pg/mL)	Lower limit of quantification (pg/mL)	Upper limit of quantification (pg/mL)	Manufacturer

TNF-*α*	1.6	15.6	1000	R and D Systems
CXCL8	3.5	31.3	1000	R and D Systems
TNFR1	50	50	45000	Invitrogen
TNFR2	100	100	138000	Invitrogen

Gene symbol (TaqMan*™*)	Assay-on-demand number	NCBI RefSeq transcript number	Amplicon length	Manufacturer

GAPDH	Hs99999905_m1	NM_002046.5	122 bp	Life Technologies
TNF-*α*	Hs00174128_m1	NM_000594.3	80 bp	Life Technologies
CXCL8	Hs00174103_m1	NM_000584.3	101 bp	Life Technologies
IL-1*β*	Hs01555410_m1	NM_000576.2	91 bp	Life Technologies
IL-6	Hs00985639_m1	NM_000600.3	66 bp	Life Technologies
TGF-*β*	Hs00998133_m1	NM_000660.4	57 bp	Life Technologies
IL-10	Hs00961622_m1	NM_000572.2	74 bp	Life Technologies

Details of blocking and flow cytometry antibodies. ELISAs and TaqMan gene expression assays are supplied.

**Table 2:** Effect of TNF-*α* mAb on pro- and anti-inflammatory cytokine mRNA expression in LPS-stimulated monocytes.

	Area under the curve (AUC) ΔCT. hours(median,range)					
	TNF-*α*	CXCL8	IL-1*β*	IL-6	IL-10	TGF-*β*
LPS	**159** (86–233)	**3050** (2170–3697)	**1571** (1436–1891)	**242** (120–1022)	**11 **(8–13)	**33** (21–50)
LPS and TNF-*α* mAb	**152** (54–197)	**2248** (1679–2988)	**940** (885–1394)	**144** (93–235)	**6** (4–10)	**29** (18–49)
*p* value		0.03	0.03	0.03	0.03	

The table outlines area under the curve values for time course profiles of each of the cytokines in response to LPS or LPS stimulation in the presence of TNF-*α* mAb (*n* = 4). AUC values were compared with a Wilcoxon Signed-Rank Test. *p* values are one-tailed.

**Table 3:** Expression patterns of TNFR1 and TNFR2 on freshly isolated monocytes.

Freshly isolated monocytes	TNFR1 only (%)	TNFR2 only (%)	Both receptors (%)	Neither receptor (%)
Subject one	0	76	1	22
Subject two	9	10	45	36
Subject three	1	72	20	7
Subject four	4	42	42	12
Subject five	6	4	87	3
Subject six	14	1	84	1

The table shows the expression patterns of TNFR1 and TNFR2 on freshly isolated monocytes from six subjects.

## Abbreviations

*μ*g: Microgram
AUC: Area under the curve
BSA: Bovine serum albumin
CD: Cluster of differentiation
cDNA: Complementary deoxyribonucleic acid
CXCL8: Interleukin 8
EDTA: Ethylenediaminetetraacetic acid
ELISA: Enzyme-linked immunosorbent assay
FITC: Fluorescein isothiocyanate
GAPDH: Glyceraldehyde 3-phosphate dehydrogenase
Ig: Immunoglobulin
IL: Interleukin
IQR: Interquartile range
LPS: Lipopolysaccharide
mAb: Monoclonal antibody
(m)RNA: (Messenger) ribonucleic acid
mL: Millilitre
MFI: Median fluorescence intensity
ng: Nanogram
PBMC: Peripheral blood mononuclear cell
PBS: Phosphate buffered saline
PCR: Polymerase chain reaction
PE: Phycoerythrin
pg: Picogram
RPMI: Roswell Park Memorial Institute
TGF-*β*: Tumour growth factor-beta
TNF-*α*: Tumour necrosis factor-alpha
TNFR1/2: Tumour necrosis factor-alpha receptor one/two.
